# Influenza A (H1N1)pdm09 outbreak of unknown source in a Ghanaian senior high school

**DOI:** 10.1186/s12889-020-09467-x

**Published:** 2020-09-18

**Authors:** Donne Kofi Ameme, Dora Dadzie, Franklin Asiedu-Bekoe, Elijah Paa Edu-Quansah, Basil Benduri Kaburi, Oxygen Wullar, Patrick Amo-Mensah, Ernest Kenu

**Affiliations:** 1 Ghana Field Epidemiology and Laboratory Training Programme, School of Public Health, University of Ghana, Accra, Ghana; 2 Ghana Health Service, Accra, Ghana; 3 University of Ghana School of Public Health, Accra, Ghana

**Keywords:** Influenza A, Outbreak, School closure, Control, Investigation

## Abstract

**Background:**

Influenza is an acute viral respiratory tract infection caused by influenza virus and transmitted from person to person. Though usually seasonal in temperate climates, influenza occurs throughout the year in the tropics with outbreaks occurring at irregular intervals. On February 6, 2018, a number of students from a Senior High School (SHS) in Accra reported to a district hospital with cough, fever and other respiratory symptoms. An influenza-like illness (ILI) outbreak was suspected. We investigated to determine the magnitude and source of the outbreak and implement control and preventive measures.

**Methods:**

We interviewed health workers, staff and students of the school as well as case-patients and reviewed health records to collect data on demographic characteristics, signs and symptoms, date of illness onset and outcome. We defined ILI case as “any person in the SHS with fever (measured axillary temperature of ≥ 37.5 °C or history of fever) and cough with or without sore throat or runny nose from January 21 to February 26, 2018”. We conducted active case search to identify more cases and took oropharyngeal samples for laboratory testing. We performed descriptive and inferential analysis by calculating attack rate ratios (ARR) and their exact 95% confidence intervals (CI).

**Results:**

Of the 3160 students, 104 case-patients were recorded from January 25, 2018 to February 13, 2018 (overall attack rate of 3.3%). Mean age of case-patients was 16.1 (±2.3) years with males constituting 71.2% (74/104). Sex specific attack rates were 5.6% (74/1331) and 1.6% (30/1829) for males and females respectively. Compared to females, males were 3.4 times as likely to be ill [ARR =3.4, 95%CI = (2.23–5.15)]. Nine oropharyngeal samples from 17 suspected case-patients tested positive for influenza A (H1N1)pdm09.

**Conclusion:**

Outbreak of influenza A (H1N1)pdm09 occurred in a SHS in Accra from January to February, 2018. Even though source of the outbreak could not be determined, prompt case management and health education on hand and personal hygiene as non-pharmacological factors probably contributed to the outbreak control. The outbreak ended with a scheduled mid-term break. This underscores the need for more evidence on the effect of school closure in influenza outbreak control.

## Background

Influenza, a respiratory transmissible infection caused by influenza virus types A, B, C and D, remains a major public health problem globally. However, only influenza A, B and sometimes C are currently known to cause respiratory infections in humans [1]. The illness can occur in individuals of all age groups but disproportionately affects persons at high risk including pregnant women, elderly persons with chronic medical conditions as well as children particularly in crowded settings such as schools [1–3]. Though generally a self-limiting illness [4], influenza can cause severe illness and death particularly among high risk populations. Globally, an estimated 3 to 5 million cases of severe illness and between 290,000 to 650,000 deaths occur annually as a result of influenza [1].

Influenza A (H1N1)pdm09 virus which was responsible for the 2009 pandemic and subsequently replaced seasonal influenza A (H1N1) as the circulating influenza A subtype [1] has caused markedly high disease burden among children worldwide [5].

Similar to other tropical countries, influenza occurs throughout the year in Ghana with epidemics occurring at irregular intervals. Since first recorded in Ghana in October 2009 with initial cases among persons with recent history of travel outside Ghana [6], influenza A (H1N1)pdm09 outbreaks have been recorded in Senior High Schools (SHSs) [6, 7] with one of the most recent outbreaks recording four deaths (case fatality rate of 5.2%). These outbreaks are usually detected via implementation of surveillance for influenza-like illnesses (ILI), which are medical diagnoses of possible influenza or other respiratory illnesses with similar symptoms.

On February 6, 2018, a cluster of acute respiratory illness cases suspected to be ILI outbreak was reported among students of a SHS in Accra. A number of the students had reported to the district hospital with cough, fever and other respiratory symptoms. A physician at the hospital noticed a rise in the number of fever and cough cases among students of the SHS presenting to the hospital. Upon notifying the hospital public health unit, oropharyngeal samples were taken from the suspected case-patients to the National Influenza Centre (NIC) for testing. On February 9, 2018, immediately after getting information about the suspected outbreak, a team of local health officials and residents and alumni of the Ghana Field Epidemiology and Laboratory Training Program joined the hospital authorities to establish the existence of the outbreak.

After initial assessment and preparations, we investigated the outbreak to determine its magnitude, identify its source and implement control and preventive measures.

## Methods

### Outbreak setting

The outbreak occurred in a SHS in the heart of Ghana’s capital city, Accra. The school has a population of 3160 students comprising 1331 (42.1%) males and 2816 (89.1%) students in the boarding houses. The school has 17 boarding houses comprising 9 female-only and 8 male-only houses. The school has a five-bed infirmary, which is manned by a physician assistant, a medical doctor and four nurses from the district hospital. The infirmary serves as the first port of call for students who are ill. Illnesses beyond the scope of the infirmary are referred to the district hospital which serves as one of the four sentinel sites for influenza surveillance in the Accra metropolis.

### Descriptive epidemiology

We interviewed case-patients who were still on admission and reviewed records at the hospital and the school infirmary to collect data on the case-patients. Data was collected from February 9 to 26, 2018 using data collection tool developed by the investigation team [see Additional file 1]. Data abstracted included age, sex, house, class, date of illness onset, date of presentation at health facility, signs and symptoms and outcomes. Based on the initial line list, all case-patients with the exception of a 35-year old nurse at the infirmary were students of the SHS who all presented with fever and cough in addition to other symptoms. We therefore defined a suspected case as “any person from the SHS with fever (measured axillary temperature of ≥ 37.5 °C or history of fever) and cough with or without sore throat or runny nose from January 21 to February 26, 2018”. Using the case definition, we conducted active case search in the hospital and school. We interviewed hospital and school staff members as well as students to identify other cases. We updated the line list with the new case-patients identified. Interviews were conducted from February 9 to 14, 2018.

### Laboratory investigations

Oropharyngeal swabs were taken from 17 case-patients, placed in a virus transport medium and transported in a cold box with ice packs at 4 °C to the NIC for testing. Complete blood count and blood film microscopy for malaria parasites were done for case-patients who were on admission at the hospital, to rule out malaria and other causes of fever.

### Environmental assessment

We conducted an environmental assessment of the school to identify possible factors for the occurrence of the cases. We assessed the boarding houses for size of dormitories, ventilation, number of windows per dormitory, number of students in a dormitory and water supply and availability of hand washing facilities. We also assessed the general sanitation of the school compound.

### Data analysis

We performed descriptive analysis of the outbreak data by person, place and time. We expressed categorical variables as frequencies and relative frequencies. Continuous variables were expressed as mean and standard deviation. We calculated the overall, sex and house specific attack rates, drew an epidemic curve and constructed a spot map representing the distribution of the case-patients in the school. We calculated attack rate ratios (ARR) by comparing attack rate between males and females as well as between the different houses. Statistical significance was determined using the exact 95% confidence intervals (CI).

## Results

### Distribution of cases by person

A total of 104 case-patients were recorded from January 25, 2018 to February 13, 2018 of which 71.2% (74/104) were males. The overall attack rate was 3.3% (104/3160) with no death. Sex specific attack rates were 5.6% (74/1331) and 1.6% (30/1829) for males and females respectively. Four (3.8%) of the case-patients were hospitalized.

**Fig. 1:**
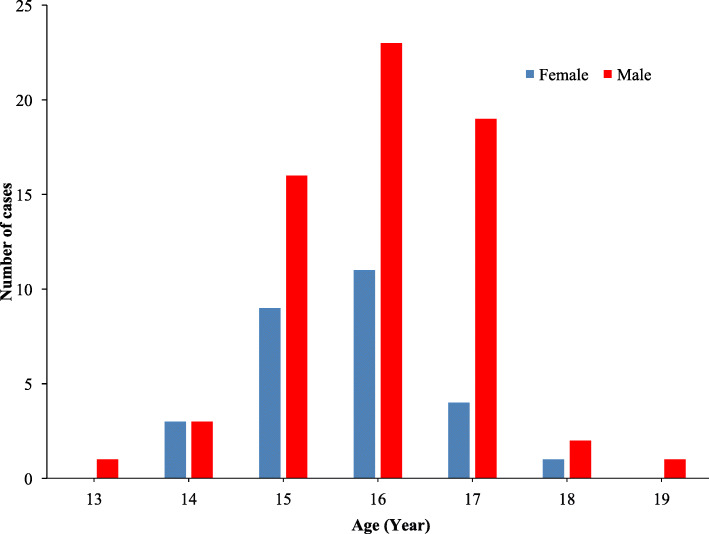
Age and sex distribution of influenza cases, Secondary High School, Accra, January–February 2018

The mean age of case-patients was 16.1 (±2.3) years and the age group with the highest proportion of cases was 15–17 years. There were more male case-patients in all ages except 14 years (Fig. 1).

With the exception of a 35-year-old female nurse at the school’s infirmary whose symptoms met the case definition, all the case-patients were students of the SHS.

The index case was a 17-year-old male form three student who reported to the school’s infirmary on January 25, 2017 with history of fever, cough and chest pain which started that same day. He had temperature of 37.3 °C on the day of presentation at the infirmary. He was diagnosed of upper respiratory tract infection and treated with antibiotics on outpatient basis. Two weeks later he presented at the district hospital with a history of fever, chills, cough, vomiting and throat pain of 3-days duration. His temperature was 39.0 °C on admission. He was diagnosed as acute tonsillitis and managed on intravenous cefuroxime, oral paracetamol and vitamin C and discharged after 3 days on oral antibiotics (cefuroxime).

Records review at the school’s infirmary showed that from January 22, 2018; there had been an average of two respiratory illnesses reported per day till on January 29, 2018 when the number of respiratory cases rose to 21. Almost all 18 (85.7%) the 21 cases were diagnosed as upper respiratory tract infection and were treated with antibiotics. Since then the number of reported respiratory illnesses had progressively increased.

All cases had fever and cough as per the case definition. Fifty (48.1%) reported runny nose, 45 (43.3%) sore throat and 44 (42.3%) chest pain (Table 1).

**Table 1:** Frequency distribution of symptoms experienced by case-patients, influenza A (H1N1)pmd09 outbreak, Senior Secondary School, Accra, January–February 2018

Symptom	Frequency	Percentage (%)
Fever	104	100.0
Cough	104	100.0
Runny nose	50	48.1
Sore throat	45	43.3
Chest pain	44	42.3
Headache	33	31.7
Chills	16	15.4
Muscle ache	9	8.7

### Distribution of suspected ILI cases by time

**Fig. 2:**
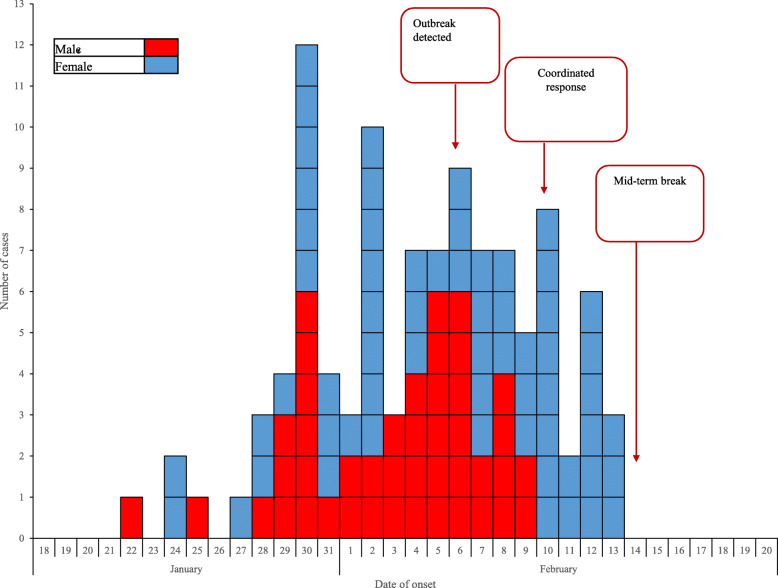
Date of onset of influenza cases, SHS, Accra, January–February 2018

A propagated outbreak commencing on January 22, 2018 with multiple peaks was observed (Fig. 2). The epidemic curve shows case-patients for whom we had date of onset of their symptoms. The general pattern is that of declining peaks from January 30, 2018 with irregular interval between peaks.

### Distribution of suspected ILI case-patients by place (boarding house)

**Fig. 3:**
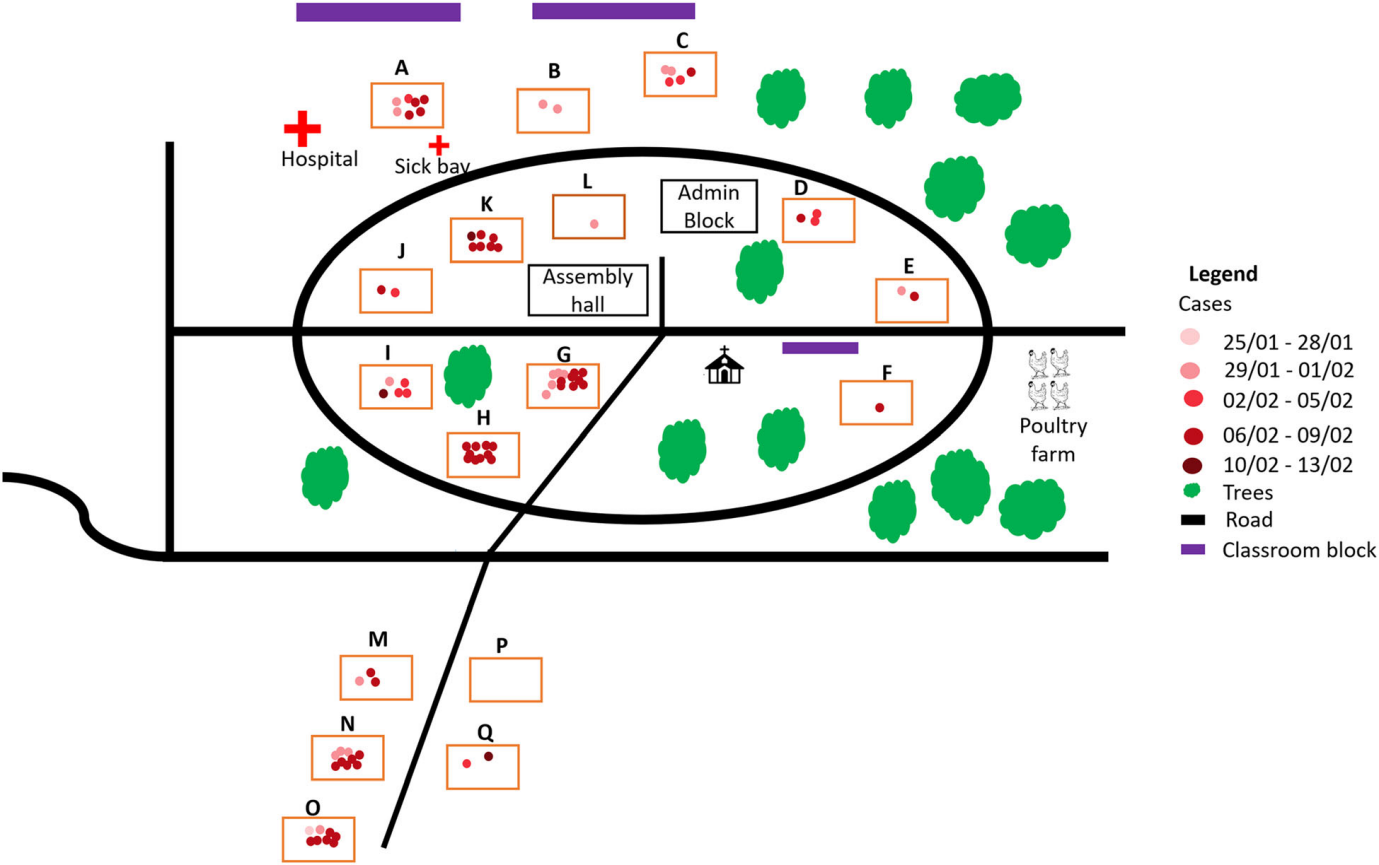
Distribution of influenza cases by house of residence, SHS, Accra, January–February 2018

House G was the worst affected with an attack rate of 8.2% followed by house H and house N with attack rates of 6.5 and 5.0% respectively (Fig. 3).

On average, the students spend 12–14 h daily in their boarding houses. However, students from all houses meet and interact in the classrooms, dinning and assembly halls.

Compared to females, males were 3.4 times as likely to be ill [ARR = 3.4, 95% CI = (2.23–5.15)] Compared to House G, houses that were less likely to have ill students include Houses B and D [ARR = 0.2, 95% CI = (0.04–0.73)], House F [ARR = 0.1, 95% CI = (0.01–0.65)], House J [ARR = 0.1, 95% CI = (0.03–0.64)], House L [ARR = 0.1, 95% CI = (0.01–0.58)] and House Q [ARR = 0.2, 95% CI = (0.04–0.67)], at statistically significant level (Table 2).

**Table 2:** Overall, sex and house specific attack rates for influenza A (H1N1) pdm09 in a SHS, Accra, Ghana, 2018

Variable	Attack Rate (%)	Attack Rate Ratio (95% Confidence Interval)	*p*-value
**Sex**			
Males	74/1331 (5.6)	**3.4 (2.23–5.15)**	**< 0.001**
Females	30/1829 (1.6)	Reference	
**House**			
House A	7/218 (3.2)	**0.4 (0.16–0.96)**	**0.034**
House B	2/145 (1.4)	**0.2 (0.04–0.73)**	**0.006**
House C	5/185 (2.7)	**0.3 (0.12–0.91)**	**0.023**
House D	2/145 (1.4)	**0.2 (0.04–0.73)**	**0.006**
House E	3/136 (2.2)	**0.3 (0.08–0.93)**	**0.024**
House F	1/143 (0.7)	**0.1 (0.01–0.65)**	**0.002**
House G	13/159 (8.2)	Reference	
House H	10/154 (6.5)	0.8 (0.36–1.76)	0.568
House I	5/152 (3.3)	0.4 (0.15–1.10)	0.065
House J	2/168 (1.2)	**0.1 (0.03–0.64)**	**0.003**
House K	7/149 (4.7)	0.6 (0.23–1.40)	0.216
House L	1/158 (0.7)	**0.1 (0.01–0.58)**	**0.001**
House M	3/153 (2.0)	**0.2 (0.07 0.83)**	**0.013**
House N	9/180 (5.0)	0.6 (0.27–1.39)	0.236
House O	7/211 (3.4)	**0.4 (0.17–0.99)**	**0.041**
House P	0/201 (0.0)	*	*
House Q	2/159 (1.3)	**0.2 (0.04–0.67)**	**0.004**

*Attack rate is 0

### Surveillance

Since the district hospital served as one of the four influenza sentinel sites in the Accra metropolis, surveillance for influenza had been on-going at the hospital. The school’s mid-term break which started on February 14, 2018 and ended on February 19, 2018 partially halted the investigation. No new cases were reported for a period of 1 week after the students returned from the mid-term break. The outbreak was confined to the school with 16 (94.1%) of the 17 houses having case-patients. Immediate community members did not have symptoms in a manner suggestive of influenza outbreak.

### Laboratory results

Of the 17 nasopharyngeal swabs tested at the laboratory, nine were positive for influenza A (H1N1)pdm09. In addition, the index case had relative lymphocytosis from the complete blood count report. None of the case-patients admitted tested positive for malaria.

### Case management

Case-patients were managed conservatively on outpatient and in-patient basis with antibiotics, antipyretics upon suspicion of primary bacterial infection or superimposed bacterial infection. Even though Tamiflu was available, it was not given because laboratory confirmation of influenza A (H1N1)pdm09 was made after the students had gone on midterm break. The delay in the laboratory confirmation was on account of lack of laboratory reagents at the time of the outbreak. All case-patients admitted had cough and temperatures above 38.^o^C. Case-patients spent an average of 3 days on admission. All nine case-patients admitted were discharged.

### Environmental issues

The school environment was generally tidy. The boarding houses spread apart on the large school compound were also generally tidy. The population of students per boarding house range from 136 to 218. On average, each house has four dormitories with an average of 38 students per dormitory. Each dormitory has eight windows measuring about one by two metres on opposite sides of the dormitory allowing for cross ventilation. The window netting on the boys’ dormitories were unclean. The students in the houses generally relied on borehole water, which was pumped at specific times. In the absence of that, students have to fetch water stored in poly tanks at the houses. The girls’ dormitories assessed were clean with good ventilation.

### Health education

Case-patients were educated on personal hygiene and ILI preventive measures such as regular hand washing with soap and water under running water and cough etiquettes prior to discharge. We also educated the entire student population and their teachers on signs and symptoms of respiratory infections as well as preventive measures including proper hand hygiene, personal hygiene and good environmental hygiene. They were also educated on social distancing measures of avoiding visiting their sick colleagues in groups and limiting other gatherings.

## Discussion

Influenza outbreaks are not new in crowded environments such as schools [8–10]. Outbreak of influenza in these and many other settings could easily go undetected or in some cases unreported. However, influenza outbreak detection in Ghana could be considered be relatively good as a result of influenza sentinel surveillance. Since its inception in 2007, Ghana’s influenza sentinel surveillance system has contributed to the detection of outbreaks as well as the circulating strains of influenza virus [11].

However, in our investigation, the outbreak had started earlier but was not detected early probably because the case-patients did not report to the health facility or health workers at the infirmary and the hospital did not have a high index of suspicion to recognize it. Seasonal influenza, because often self-limiting [4], does not usually attract critical attention from both patients and health workers alike. Oftentimes, as a common practice, case-patients with ILI will choose self-care option for initial management rather than seeking care at the health facility. This may possibly explain the late detection of the outbreak. In spite of that, coordinated efforts of responding to the outbreak yielded positive results. The epidemic curve showed a bulk of the cases prior to the date of detection of the outbreak which may have been averted had detection been done earlier. All cases that met the case definition but were not confirmed by laboratory test were considered as clinical cases and included as part of the outbreak cases.

With respect to the risk factors of the outbreak, it is unclear why males were significantly more likely to be affected compared to their female counterparts. However, what is more striking is the fact that the index case was a male and the houses with the relatively higher attack rates were male-only houses. It is likely that the illness started from the male house with person to person transmission and spread to affect females.

Regarding the outbreak control, several coordinated efforts including case management and health education were important measures. Even though school closure was not considered as a control measure in this outbreak, the school’s scheduled mid-term break played similar role as school closure. The issue of closing schools during major influenza epidemics still remains debatable with no consensus, as most studies conducted on this using different methods have either produced conflicting results or not generated enough information to answer the question [12, 13]. This is much so because the World Health Organization’s recommendations on pandemic influenza are equally based on limited data and vary depending on several factors such as the transmission pattern, and severity and extent of illness [14]. There is paucity of empirical data on effect of school closure during influenza outbreaks on illness transmission within affected schools. Most studies considered how school closure affected community-wide influenza transmission. While some of these studies observed beneficial effects of school closure on influenza outbreaks [10, 13, 15, 16], others found it to be ineffective [17]. It is however difficult to ascertain to what extent the school’s mid-term break, akin to school closure in this outbreak, contributed to ending the outbreak. This is because, like in other instances where reactive school closure was adopted as a counter measure for influenza outbreaks, it was rather late in the outbreak or implemented simultaneously with other measures [13]. It is therefore difficult to attribute the outbreak control to school closure, which could likely be a coincidence. On the contrary, school closure could not be overlooked as contributing factor to the outbreak control. It is also possible that, widespread immunity was achieved by students contracting the illness for which reason transmission did not resume when students returned from the mid-term break. Whereas it could be argued that school closure under most circumstances was studied as a measure to reduce influenza outbreak at the community level, the outbreak response by Smith et al. considered the possible effect of school closure on influenza outbreak in the affected school [10]. Also, the World Health Organization framework for national and local level planning to reduce transmission of influenza A (H1N1)pdm09 in school settings considered school closure as a key intervention. This is premised on the fact that schools play a critical role in amplifying influenza transmission both within schools and the wider community [18]. School closure during influenza outbreaks, particularly when done early in the course of the outbreak as a proactive measure, reduces illness transmission in schools and thus spread of illness into the wider community. Therefore, in as much as school closure may be considered as a measure for reducing influenza outbreak at the community level, it primarily contributes to reduction of transmission within the school itself; with the anticipated secondary effect of controlling community level outbreak [18, 19].

Influenza outbreak control in schools have been handled differently in different settings. Whilst some authors have recommended a combination of vaccination of 70% of susceptible individuals prior to outbreak and case-patient isolation as key measures for control of influenza outbreak in schools [20], others have proposed isolation as a single intervention [21] and individual school dismissal [22]. However, none of these interventions were employed in our investigation. Influenza vaccination is not part of the routine immunization under Ghana’s Expanded Programme on Immunization and therefore not given routinely as a pre-exposure prophylaxis. Reactive influenza vaccination has been considered and utilized in an influenza outbreak in a school [7] even though the use of antiviral oseltamivir for outbreak control is much more common [6, 9, 10]. It is instructive to note that, just as in our investigation, non-pharmacological interventions alone have been used to respond to influenza outbreak in a school setting [2]. Use of a suite of non-pharmacological interventions either as standalone measures or in combination with antivirals has been highly recommended for control of influenza outbreaks especially in resource-poor settings [23].

### Limitations

Our investigation had some limitations that should be considered in interpretation of the results. Firstly, as we did not follow up on the case-patients to their respective homes, we could not determine the spread of the infection in the community. Since the students went on mid-term break while the outbreak was ongoing, there was a high likelihood of them infecting their household and community contacts. However, determination of this possible secondary cases was not feasible as the students went to their homes dotted across various parts of the country. In a situation where student interactions still continued after school closure, without proper hand hygiene and respiratory etiquettes, disease transmission could still occur. We were however unable to determine if this out-of-school student contact characterized this outbreak. Secondly, we were unable to determine the source of the outbreak as it was difficult determining the primary case. The index case who first developed symptoms on January 25, 2018 was predated by some cases which could either be outbreak related or baseline cases of ILI. In spite of these limitations, determination of the aetiology and successfully instituting control and preventive measures were considered as topmost priority in our investigation and response. Another limitation worth considering is the fact that, though not all students went home during the mid-term break, we did not collect data on the proportion and characteristics of students who stayed at school during the mid-term break to understand whether the outbreak stopped because of lack of susceptible persons. Also, we did not calculate attack rate ratios by age group because data on the ages of all the students were unavailable to us.

## Conclusions

An influenza A (H1N1)pdm09 outbreak occurred in a SHS in Accra from January to February, 2018. Source of the outbreak could not be determined. Prompt case management and health education on hand hygiene, environmental hygiene and cough etiquette for members of staff and students were preventive and control measures. Scheduled mid-term break, though not the magic potion, probably had a role in this outbreak control. Additional evidence on the effect of school closure on influenza outbreak control in school settings is warranted.

## Supplementary information

**Additional file 1.** Influenza outbreak interview guide. Data collection tool used to collect data during the outbreak investigation.

## Data Availability

The dataset used during this outbreak investigation are available from the corresponding author on reasonable request.

## Abbreviations

ARR: Attack rate ratio
CI: Confidence interval
ILI: Influenza-like illness
NIC: National Influenza Centre
SHS: Senior High School
