# Supplementary material for: Influenza A (H1N1)pdm09 outbreak of unknown source in a Ghanaian senior high school
Source: BMC Public Health. 2020 Sep 18;20:1423. doi: 10.1186/s12889-020-09467-x (PMC7499409; doi:10.1186/s12889-020-09467-x)
Supplement: Supplementary file 1 — Additional file 1. Influenza outbreak interview guide. Data collection tool used to collect data during the outbreak investigation. [file 12889_2020_9467_MOESM1_ESM.pdf]

SUSPECTED OUTBREAK OF INFLUENZA AT *xxx* SENIOR HIGH SCHOOOL, ACCRA

FEBRUARY 2018

DATA COLLECTION TOOL

Name of patient	
Date of Birth (dd/mm/yy)	☐☐☐☐☐☐
Age in completed years	
Sex	Male ☐<br>Female ☐
Hall of residence	
Class	
Telephone	☐☐☐☐☐☐☐☐☐☐
Type of Student	Border ☐<br>Day Student ☐
Residential address (Town of residence)	
Travel history. If yes, probe and describe	

Date of symptom onset (dd/mm/yy)	| | | | | | |
Date of reporting (dd/mm/yy)	| | | | | | |
Signs and symptoms	Fever: ___ Cough: ___ Sore throat: ___ Other(s), Specify: ___
Temperature in °C	
Diagnosis (As by attending health worker)	
Status	Outpatient: ___ In Patient: ___
Outcome	

<u>For Index and Primary Case</u>
Ask Questions and probe to describe in detail:

- Travel history
- Participation in events eg. Party, sporting events
- Contact with ill persons
- Contact with animals
- Date of onset of symptoms
- Medications taken
- Care sought for illness

<u>For School and Health Authorities</u>

Description of the nature of the situation: Date problem identified, persons involved, measures put in place eg. Lab, treatment, etc., next steps
